# Does orthodontic treatment provide a real functional improvement? a case control study

**DOI:** 10.1186/1472-6831-13-57

**Published:** 2013-10-24

**Authors:** Chiara Masci, Irma Ciarrocchi, Alessandro Spadaro, Stefano Necozione, Maria Chiara Marci, Annalisa Monaco

**Affiliations:** 1 Department of Life, Health and Environmental Sciences, V. le Vetoio 1, L'Aquila 67100, Italy

## Abstract

**Background:**

Electromyographic analysis of the masticatory muscles provides useful data on the behavior of these muscles during stomatognathic system functioning and allows a functional assessment of orthodontic treatments. This study was undertaken to verify if achieving an Angle Class I bite through orthodontic treatment can lead to neuromuscular balance.

**Methods:**

This study enrolled 30 patients (20 females, 10 males, mean age: 15.78 years) with an Angle Class II, division 1 malocclusion that was orthodontically treated. A group of 30 subjects (19 females, 11 males; mean age: 16.15 years), randomly selected among subjects with an Angle Class II, division 1 malocclusion that had not been orthodontically treated served as the Control group. Both groups were subjected to electromyography to study their neuromuscular characteristics. The Shapiro-Wilk's test revealed a non normal distribution, therefore we used a Friedman two way ANOVA by ranks test to compare differences of surface electromyography values between treated and untreated subjects at closed and open eyes condition.

**Results:**

A statistically significant interaction between orthodontic treatment and open eyes conditions was detected for anterior temporal muscles. A significant imbalance of the anterior temporal muscles, which is indicative of an asymmetric electromyographic pattern, was also found.

**Conclusions:**

The present data indicate that achieving a correct occlusal target does not necessarily correspond to a neuromuscular balance.

## Background

The primary objective of orthodontic treatment is to achieve ideal positional relationships among the teeth within and between the arches [1-3]. Positional corrections can be made by moving the teeth and by modifying the skeletal structures and growth of the cranial and facial skeleton. An Angle’s Class I occlusion between canines and molars is considered to be the orthodontic target, in terms of both aesthetics and functionality, for patients presenting with substantial maloccusion [1,2]. The achievement of muscle balance at the end of orthodontic treatment is another very important objective often overlooked. In fact, the lack of a muscle balance could compromise the stability of the result achieved by orthodontic treatment and could require an endless use of retainers for retention [4].

In literature, several studies have investigated the achievement of a neuromuscular balance after orthodontic treatment. According to Hirata et al. [5] orthodontic treatment does not always lead to the achievement of a muscular balance, indeed they show that there is an equal prevalence of dysfunctions in patients treated orthodontically and untreated controls. Other studies have shown that patients who have undergone orthodontic treatment present more evident signs and symptoms of temporomandibular joint disorder (TMD) than subjects with malocclusion who have not been orthodontically treated [6]. Besides, recent studies suggest that it is difficult to establish a relationship between a certain type of malocclusion and TMD [7-11]. In light of the most recent literature, for the characterization of the functional aspects of the stomatognathic system, it is not sufficient to rely on the classic structural and aesthetic parameters used in orthodontics, based on cephalometric and dental class evaluations. Hence, orthodontists have in recent years started to use diagnostic instruments, such as surface electromyography (sEMG), in functional studies of the stomatognathic system [12,13]. In clinical orthodontics, electromyography has been used to evaluate the influence of occlusal conditions on the neuromuscular balance of the stomatognathic system [14-17] and to evaluate, from a functional point of view, the efficacy of orthodontic treatments [12,18-22] De Rossi et al. [12] analyzed the electromyographic activity of the masseter and temporal muscles in 27 pediatric patients (average age, 8.6 years) who were subjected to rapid maxillary expansion and found that the electromyographic activity of the examined muscles increased significantly after orthodontic treatment.

Saccucci et al. suggested that the use of a preformed functional device in subjects with Class II, division 1 malocclusion, deep bite, and labial incompetence, treated with interceptive orthodontics, induces a significant increase of the sEMG activity of the lower orbicular oris (OO) muscle at rest and of the upper OO muscle during mandibular protrusion [22].

Bothelho et al. [21] recorded the electromyographic activity of masseter and temporalis anterior muscle with the aim of assessing neuromuscular changes following orthodontic treatment, establishing that there were no statistically significant differences between the treated and no-treated subjects. Castroflorio et al. [19] assessed the effects of orthodontic functional appliance (FGB-D) on the masticatory muscles by performing sEMG in 33 young adults; they concluded that the functional appliance were effective in correcting mandibular lateral displacement. Ferrario et al. [18] attempted to quantify the influence of masticatory muscles on post-orthodontic-treatment relapses with the goal of excluding unnecessary procedures. Kecik et al. [20] compared stomatognathic changes before and after maxillary expansion treatment using a quad-helix appliance in a group of patients with a posterior cross-bite in mixed dentition, employing radiographic, clinical, and electromyographic exams. They demonstrated that diagnosis of malocclusion and assessment of results of orthodontic treatment should not be limited to clinical and cephalometric evaluations, but should also involve sEMG. sEMG allows one to formulate adequate diagnoses and prognoses and also to monitor the functional impact of orthodontic therapies over various treatment phases. There is heated discussion about the usefulness of sEMG in the study of stomatognathic system, but most reviews suggest some cautions. Indeed, sEMG’s diagnostic reliability and validity, as well as its therapeutic value, have been questioned. It is generally thought that the physiological variables that may affect the validity of sEMG are age, sex, skeletal morphology and psychological factors [23-25].

It is also important to consider the influence of the visual system on the stomatognathic system when sEMG is performed. Infact visual input play an important role in the multisensory process of postural stabilization. Ocular nuclei send fibres to the nuclei that control neck and head movements and receive afferent input from vestibular nuclei. A modification of ocular proprioception modifies head and body posture. In a study by Monaco et al. [26], electromyographic comparison between eyes closed and eyes open conditions with the mandibular in a rest position were used to show a state of neuromuscular unbalance that can affect the occlusal state of patients.

Despite the usefulness of electromyographic analyses, the neuromuscular characteristics associated with a class II, division 1 malocclusion have not been investigated using this method. Therefore, the purpose of this study was to assess the neuromuscular balance of a group of individuals with Angle class II, division 1 malocclusion who have been orthodontically treated to reach a class I molar/canine positions and overjet resolution, relative to a group of non-orthodontically-treated age- and sex-matched subjects with the same diagnosis and similar dental-skeletal characteristics as the treated group. In so doing, we sought to determine whether achievement of Angle Class I leads to a neuromuscular balance that can be verified by sEMG.

## Methods

### Study design

The aim of this study was to investigate if there were significant differences in sEMG values between II class malocclusion treated and untreated patients.

### Setting and subjects

**Figure 1:**
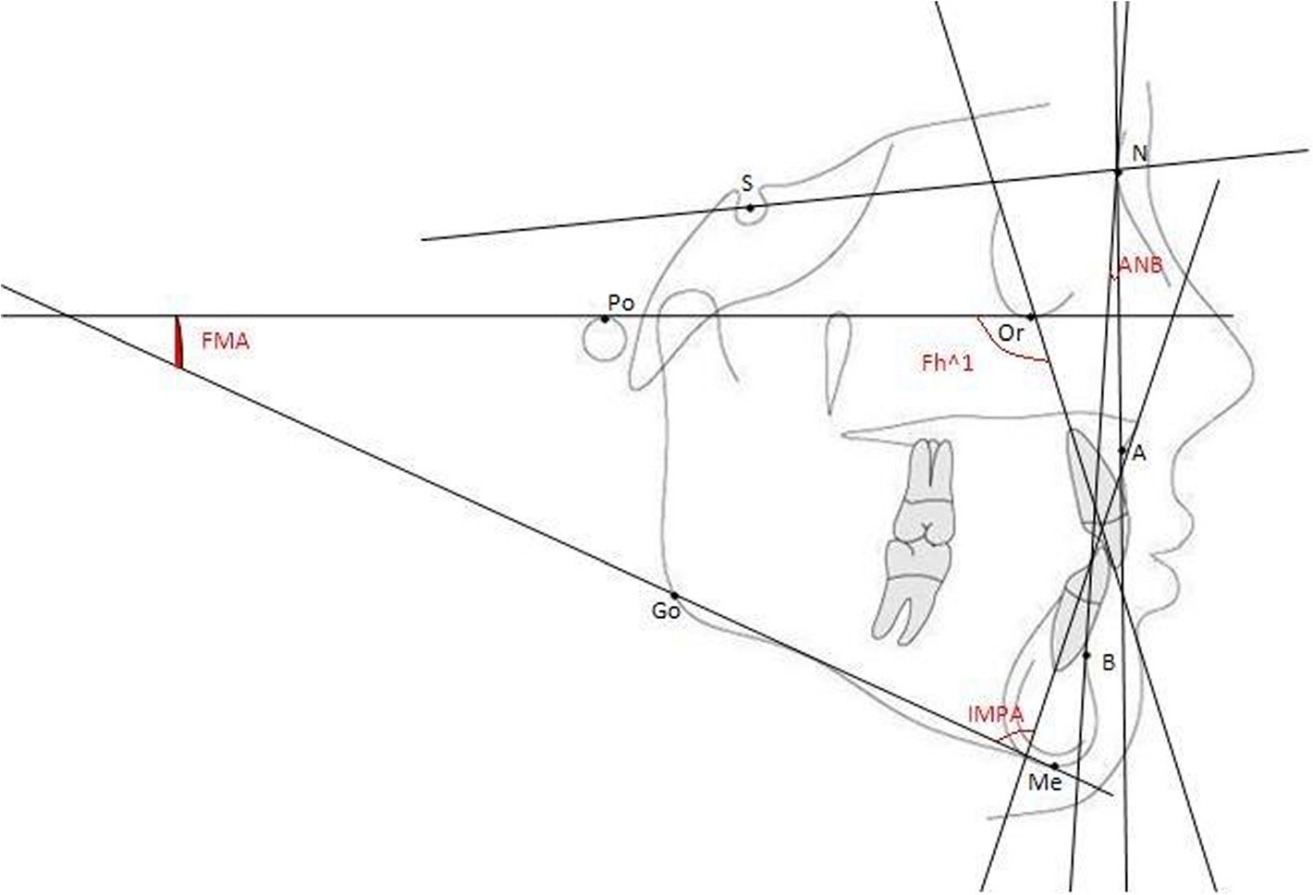
**Hard-tissue landmarks used in lateral cephalometric radiographs: nasion (Na); orbitale (Or); sella (S); porion (Po); A-point (A); B-point (B); menton (Me); gonion (Go).** Skeletal angular and linear measurements of lateral cephalograms: ANB = angle that provides information on the ralative positions of the jaws to each other and provides a genaral idea of the anteroposterior discrepancy of the maxillary to the mandibular apical bases. Fh^1 = angle that measures the inclination of the upper incisors with respect to the maxilla. FMA = angle that shows the type of facial growth of the subject. IMPA = angle that measures the inclination of lower incisors with respect to the jaw.

This study was carried out at the Dentistry Center of University of L’Aquila. This study was conducted in accordance with the declaration of Helsinki. The committee on ethics in science of the University of L’Aquila approved the study with the number 0018365/12. Participation in the study was voluntary and a written consent was obtained from the parents or guardians. From a total of 76 patients who had completed orthodontic treatment at least one year prior to the study 30 (males, n = 10; females, n = 20 mean age: 15.78 years) who met the following clinical and cephalometric criteria, based on clinical evaluation and cranial radiography (latero-lateral projection) were selected. Patients were selected according to the following inclusion criteria: molar and canine class I, 0 < overjet <4 mm, absence of facial-skeletal asymmetry, absence of tooth rotation and ANB angle within 0–4° with Fh^1 = 110° ± 4, FMA = 25 ± 10, and IMPA = 90 ± 4 (Figure 1). The exclusion criteria applied to both groups were: (1) presence of carious teeth; (2) presence of dental restorations that might alter dimensions, shape, and position of the mid-point of the clinical crown; (3) presence of prosthetic crowns or gingival defects; (4) missing teeth; (5) periodontal disease; (6) presence of marked crowding or spacing; (7) presence of a unilateral or bilateral cross-bite; (8) clinical signs or symptoms of temporomandibular clinical dysfunction; (9) trauma in the dental-facial region; (10) skeletal asymmetry; (11) genetic or congenital anomalies; (11) systemic diseases that may negatively affect growth; and (12) current or previous use of systemic drugs such as steroids. Our protocol requires the analysis of subjects with absence of visual defects [26,27].

A group of 30 sex- and age-matched subjects (males, n = 11; females, n = 19 mean age: 16.15 years) with Angle class II, division 1 malocclusion who had received no orthodontic treatment acted as Control group. The mean age and sex ratios between the Control group and the Study group did not differ significantly. The two groups before treatment were statistically homogeneous in terms of their clinical and cephalometric values.

### Orthodontic treatment

Study group patients included were subjected to orthopedic therapy during the growth peak to obtain a skeletal balance in the sagittal and transversal planes and to promote mandibular advancement in the sagittal plane because, in second class, this bone structure is usually retropositioned.

They were subsequently subjected to finalization with non-extraction orthodontic treatment with fixed appliances multibrackets and use of Class II elastics to achieve good alignment of the teeth and to reach Andrews’ six occlusion keys [2].

### sEMG

Two electromyographic recordings were obtained for each subject while in a rest position (teeth not in contact), seated on a wooden chair with a straight back in a comfortable room. During the first recording, each subject was asked to keep his/her eyes closed. During the second recording, each subject was asked to open his/her eyes and to look straight forward, maintaining light contact between his/her lips. The participants received these instructions before commencement of recordings. The ambient light was a standard hospital illumination. The windows were obscured. A researcher observed the patient's face to control if the children had unwanted motion, and eventually repeated the examination. The recording duration for each sEMG test was 15 seconds. Electromyographic activity was recorded with an eight-channel K7 system (Myotronic Inc.; Seattle, WA) using pre-gelled adhesive surface bipolar electrodes with an inter-electrode distance of 20 mm. The skin surface where the electrodes were applied was cleaned before placement of electrodes. Electrodes were positioned on the left and right masseter muscles (LMM, RMM) and the left and right anterior temporal muscles (LTA, RTA), as described by Castroflorio et al. [28,29], as well as on the left and right anterior digastric muscles (RDA, LDA) [30] and the left and right sternocleidomastoid muscle (LSC, RSC) bilaterally parallel to the muscular fibers and over the lower portion of the muscle according to Falla et. al [31] to avoid innervations point. A template was used to enabled the electrodes to be re-positioned in the same position when the measurements were repeated at different times or if an electrode had to be removed because of a malfunction.

Electrical signals were amplified, recorded, and digitized using software designed for clinical purposes (K7, Myotronics Inc., Seattle, WA). Root mean square (RMS) values (in μV) were used as indices of the signal amplitude.

An expert examiner, who was not informed of the purpose of the study, carried out the sEMG recordings. The data were analyzed by a second investigator, who was also uninformed about the reasons for the analysis and was blind to the group designations of the subjects.

The repeatability of the recording protocol was investigated for the test conditions, by asking the selected subjects to repeat the sEMG recording two times, with a gap of 15 min between the two recordings. The results of the first and second set of experiments showed a repeatability of measurements.

To ensure anonymity, each subject was randomly assigned an alphanumeric code. To reduce the possibility of bias, groups assignments were not revealed until the data were compared.

### Statistical analysis

The average values of root mean square (RMS) of the tracks performed with closed eyes were compared with the averages of EMG performed with open eyes. Repeatability of assessments had been previously tested on a set of 30 patients with the Intraclass Correlation Coefficient (ICC), which had reported values ranging from 0.9668 (CI = 0.9404-0.9829) and 0.9886 (CI = 0.9792-0.9941). The ICC values obtained for the examined muscles shows an excellent repeatability [32].

The distribution of the electromyographic data was analysed by Shapiro-Wilk test that revealed that data were not normally distribuited. For this reason data were analyzed with a Friedman two way ANOVA by ranks test to compare differences of sEMG values between treated and untreated subjects at closed and open eyes condition. The level of significance was assumed at the value of p ≤ 0.05.

From the electromyographic data, symmetry index (SI) values were calculated as described by Humsi et al. [33], according to the formula (a - b)/(a + b), where a and b represent the values of the homologous muscles of each muscle in the compared conditions. Shapiro-Wilk test revealed that the symmetry index (SI) values were normally distributed. Therefore, a paired t-test for dependent samples was used to compare SI values in the eyes closed versus the eyes open conditions, and paired t-tests for independent samples were carried out to compare SI data within each condition (open or closed eyes). The first null hypothesis posited that pattern would not differ between the two conditions. If there were significant differences between open and closed eyes condition in SI data the null hypothesis was rejected and the alternative hypothesis that visual input affect SI would be supported.

Subsequently, we conducted paired t-tests for independent samples to compare closed and open eyes conditions between the two groups. Thus, the second null hypothesis posited that the two groups would not differ from each other in either condition, such that the SI values of the Control and Study groups behave in homogeneous way within one or in both conditions. Alternatively, if the groups’ SI values do differ from each other in one or in both conditions, the null hypothesis would be rejected, and the alternative hypothesis that orthodontic treatment affects SI would be supported. P values less than or equal to 0.05 were considered significant and indicated that the null hypotheses should be rejected in favor of the alternate predictions.

Statistical analyses were performed with SAS version 9.2 (2008; SAS Institute Inc.).

## Results

### Study cohort demographics

As shown in Table 1, of the mean age and sex ratios did not differ significantly between the Control and Study groups.

**Table 1:** Group comparisons of means (standard deviations) of demographic data

**Parameter**	**Control group**	**Study group post-tx**	**P**
Age (years)	16.15 (1.26)	15.78 (1.03)	NS
Sex	19 females, 11 males	20 females, 10 males	NS

NS, not significant (p > .05); tx, treatment.

### Cephalometric data

The mean values of the cephalometric data of the two groups are compared in Table 2. The cephalometric data for the Study group before treatment were similar to the Control group data. However, significant differences were found for all parameters between the Study group after treatment dataset versus the Control group and the Study group before treatment datasets, with the exception of the comparison between pre-treatment Study group versus post-treatment Study group for overbite.

**Table 2:** Analysis of the effects of treatment on cephalometric data, means (standard deviations)

**Parameter**	**V.N.**	**Study group pre-tx**	**Control group**	**P value: pre-tx vs. Control**	**Study group post-tx**	**P: control vs. post-tx**	**P: pre-tx vs. post-tx**
ANB	0–4°	6.6 (1.3)	6.4 (1.2)	0.19	3.7 (0.69)	**0.001**	**0.001**
Fh^1	106–114°	115.9 (2.8)	115.2 (2.4)	0.33	108.3 (2.3)	**0.001**	**0.001**
FMA	15–35°	25.5 (2.0)	25.4 (2.5)	0.44	28.5 (2.3)	**0.001**	**0.001**
IMPA	86–94°	88.4 (2.06)	89.4 (2.34)	0.10	91.5 (1.9)	**0.001**	**0.001**
Overjet	0–4 mm	6.6 (1.3)	6.5 (1.3)	0.45	3.05 (0.91)	**0.001**	**0.001**
Overbite	0–4 mm	2.6 (1.0)	2.5 (1.1)	0.38	3.2 (1.1)	**0.04**	0.06

Significant p values in bold.

### sEMG and SI

**Figure 2:**
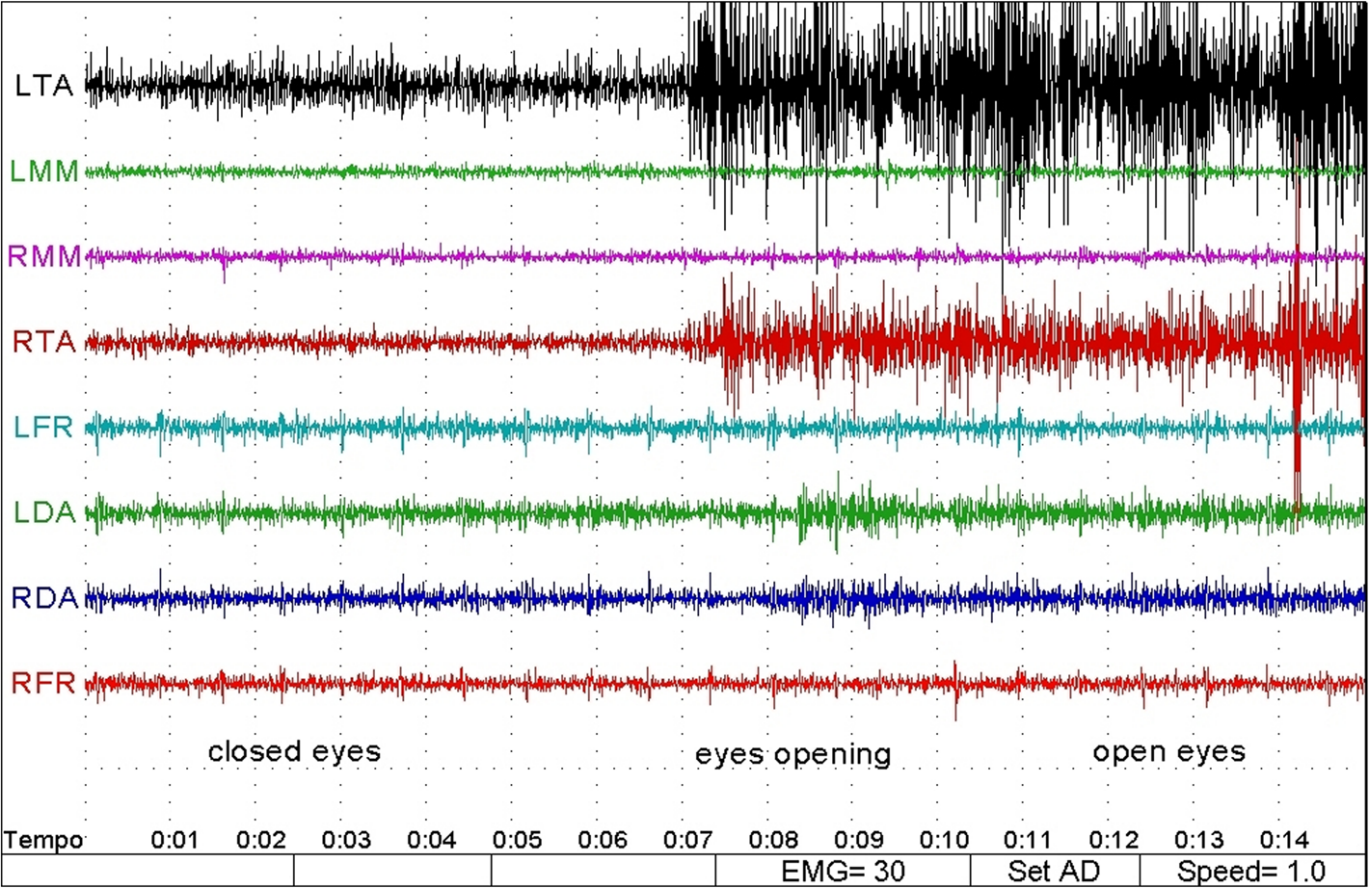
**SEMG track for a patient of the treated group.** RTA and LTA have an higher activation in the opening and the open eyes condition than closed eyes.See text for abbraviations. The numbers on the right of the tracks represent the RMS in microvolts for each muscle.

**Figure 3:**
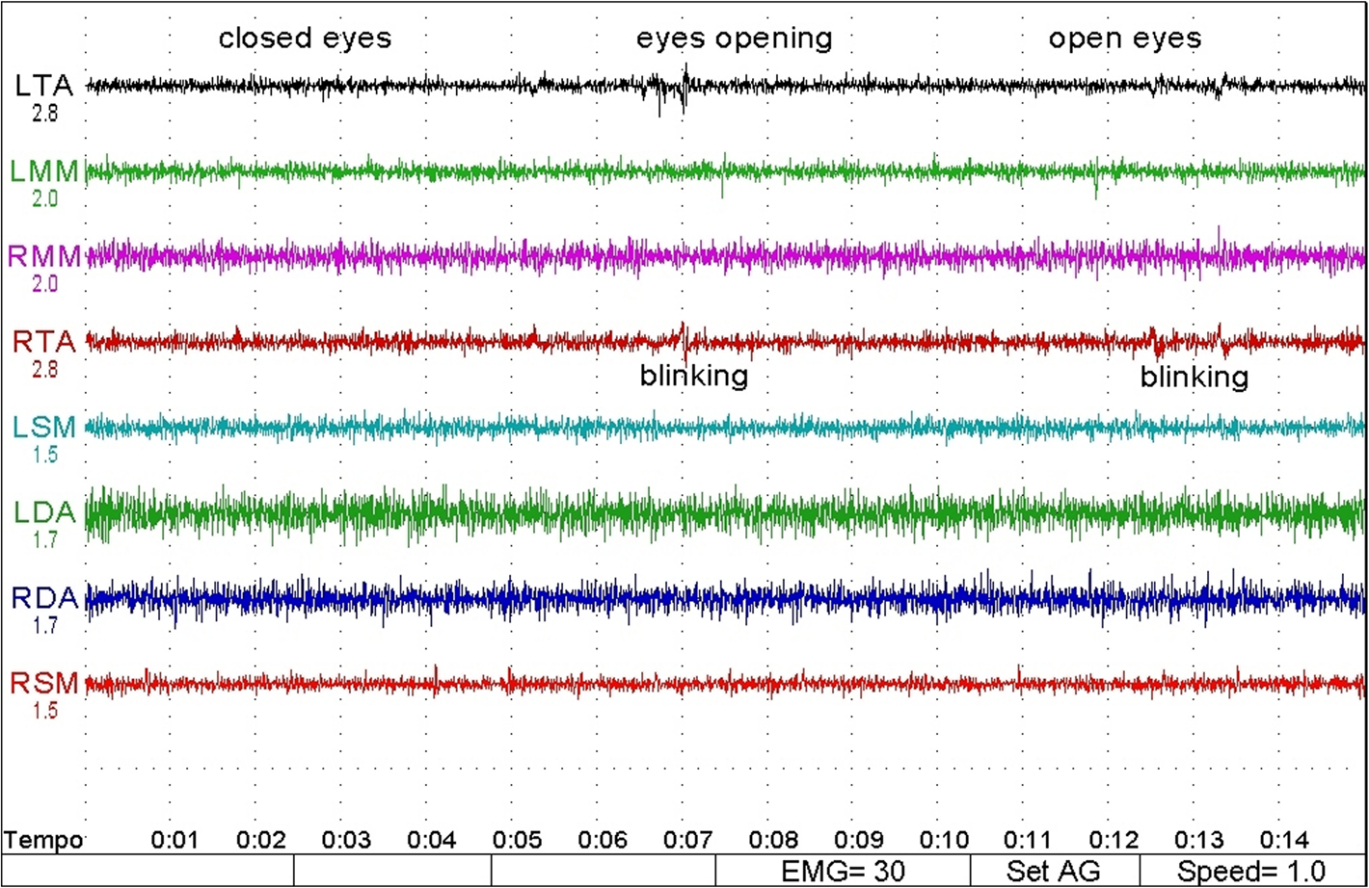
**SEMG track for a patient of the control group.** No differences in sEMG activity of the muscles between open and closed condition. See text for abbraviations. The numbers on the right of the tracks represent the RMS in microvolts for each muscle.

**Figure 4:**
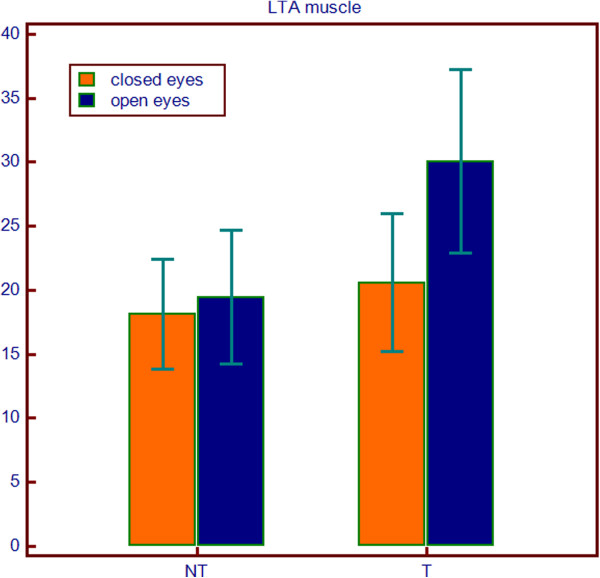
Differences of EMG value of LTA muscles between study and control groups in closed and open eyes conditions.

**Figure 5:**
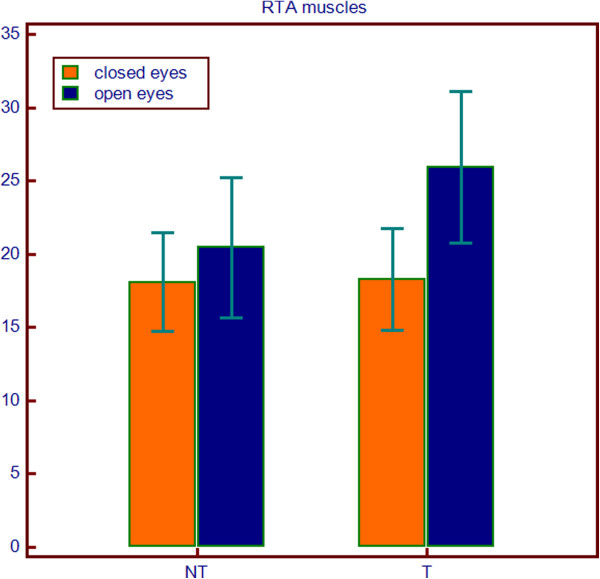
Differences of EMG value of RTA muscles between study and control groups in closed and open eyes conditions.

Friedman's analysis showed a significant difference between orthodontic treatment and open eyes conditions for variables LTA (p = 0.0398) and RTA (p = 0.0246), while for the other variables has not been demonstrated any interaction between treatment and eyes conditions. (Table 3) (Figures 2, 3, 4 and 5).

**Table 3:** Significance levels of differences in sEMG values between study and control groups in investigated muscles at open eyes condition (Friedman’s test)

**Muscles**	**Mean values control group**	**Mean values study group**	**P VALUE**
LTA	3.01 ± 1.91	1.95 ± 1.4	**0.0398**
RTA	2.59 ± 1.39	2.05 ± 1.28	**0.0246**
LMM	1.48 ± 0.94	1.29 ± 0.84	0.8085
RMM	1.24 ± 0.6	1.12 ± 0.61	0.1358
LDA	1.91 ± 0.97	2.03 ± 1.14	0.2305
RDA	2.08 ± 1.22	1.9 ± 1.04	0.1956
LSC	2.18 ± 1.51	1.6 ± 1.04	0.9226
RSC	2.07 ± 1.28	1.71 ± 1.15	0.2119

Significant p values in bold.

None of the SI values of homologous muscles differed between the open eyes versus closed eyes conditions (Table 4) within each group. Meanwhile, only comparison between groups revealed a significant difference. That is, in the open eyes condition, the anterior temporal muscles had a higher SI value in the Study group after treatment than in the Control group. All other SI values in both conditions did not differ significantly between the groups.

**Table 4:** Muscle SI (standard deviation) comparisons between conditions and between groups

**Group**	**TAMs**		**P**	**DAMs**		**P**	**SCMs**		**P**	**MMs**		**P**
	**Closed eyes**	**Open eyes**		**Closed eyes**	**Open eyes**		**Closed eyes**	**Open eyes**		**Closed eyes**	**Open eyes**	
Study, post-tx	0.18 (0.15)	0.20 (0.13)	NS	0.11(0.10)	0.12 (0.09)	NS	0.16 (0.13)	0.15 (0.14)	NS	0.17 (0.16)	0.17 (0.15)	NS
Control	0.17 (0.15)	0.14 (0.12)	NS	0.11 (0.09)	0.10 (0.08)	NS	0.17 (0.15)	0.14 (0.13)	NS	0.14 (0.11)	0.12 (0.11)	NS
P	NS	<.01	-	NS	NS	-	NS	NS	-	NS	NS	-

TAMs, Anterior temporal muscles; DAMs, Anterior digastric muscles; SCMs, Sternocleidomastoid muscles; MMs Masseter muscles.

## Discussion

This study evaluated the functional balance of subjects without visual defects with class II division 1 malocclusion who had or had not been subjected to orthognatodontic treatment aimed at achieving a molar and canine Angle class I. For this aim, sEMG was conducted on four pairs of muscles (Anterior Temporalis, Masseter, Anterior Digastric, and Sternocleidomastoid) at rest. Various authors have underscored the need for functional evaluation of the stomatognathic system [12,34,35]. Conventional orthodontic treatment aims to achieve morphological and aesthetical norms based on static assessments. However, the functioning of the cranial-mandibular muscles and of the joints involved with occlusion occurs through interactions with the nervous system. Hence, it would be desirable to employ an approach that assesses functional aspects of the occlusion, unlike conventional malocclusion diagnosis techniques [36].

Several recent studies have verified the muscular features of patients subjected to orthodontic treatment and orthognatic surgery. In particular, using sEMG, Botelho et al. [21] in their cross-sectional study analyzed the neuro-muscular changes in maximum voluntary clench (MVC) that take place following orthodontic treatment comparing subjects who were undergoing orthodontic intervention with subjects who had no orthodontic intervention. In the cross-sectional study of Tartaglia et al. [37] the effects of functional orthodontic devices on muscular function was evaluated in MVC comparing orthodontic patients with healthy subjects who were not orthodontically treated. A longitudinal study of Nuño-Licona et al. [38] analyzed the electromyographic muscular patterns in MVC of 10 children with a class III malocclusion before, during, and after treatment with myofunctional appliance (the monobloc). The authors demonstrated that sEMG is a non-invasive method, extremely useful for studying functional effects of the orthodontic treatment. The longitudinal study of Van den Braber et al. [39] demonstrated that electromyography can be used to study the effects of orthognatic surgery on masticatory function in patients with mandibular retrognathism. In this study the electromyographic activity (EMG) was performed during isometric clenching and during chewing. These studies concluded that the treatments did not significantly affect electromyographic values, indicating that the treatments did not significantly alter, positively or negatively, the neuromuscular condition of the patients.

The aforementioned studies compared treated versus non-treated groups without specifying whether the subjects eyes were open or closed during testing, and thus without any analysis on the influence of visual stimulation on electromyographic data [40].

The influence of visual input on stomatognatic muscles is well known [40]. However, only a recent study [27] have shown that in healthy subjects there are no differences in the sEMG activity of anterior temporal muscles at open and closed eyes condition, while in disfunctional subjects, the sEMG activity of anterior temporal muscles is higher in open eyes condition than in closed eyes one, suggesting that sEMG testing could be employed to reveal or confirm a dysfunctional condition [26].

Furthermore, there have been a small number of studies that have used the SI [19,33,41], suggesting that symmetry in electromyographic values should be considered as an index of normal muscular function.

Therefore, this study considers the effects of visual input on the stomatognathic system (open and closed eyes conditions) and it evaluates symmetry index (SI) for testing symmetry between homologous muscles. Our results show that, treated subjects showed an increase in electromyographic values of the RTA and LTA when they transitioned from closed eyes to open eyes. Furthermore, the present data also suggest an exacerbation of asymmetry when the eyes are opened that is indicative of a worsening of neuromuscular function, at least under a rest condition [42]. Hence, we can deduce that although orthodontic treatment in subjects with class II division 1 malocclusion toward an Angle class I bite (meeting all 6 Andrews’ keys) [2] can bring an aesthetic occlusal result, it is not necessarily accompanied by a neuromuscular balance at rest. Meanwhile, these phenomena were not observed in the Control group, suggesting that the impairment in the neuromuscular balance observed in the Study group may not be strictly associated with the presence of a class II, division 1 malocclusion [43].

Our observation that subjects at rest who had been treated orthodontically for a class II, division 1 bite did not have neuromuscular balance that was on par with Control subjects supports prior suggestions that there are a large number of patients in the orthodontically treated population that continue to have neuromuscular dysfunction [44,45]. Hence, more attention should be paid to neuromuscular diagnosis in subjects who are being prepared for orthodontic treatment so that orthodontists might better understand the reasons underlying failures and relapses [46]. This suggestion is in agreement with Wang et al. [6] that patients subjected to orthodontic treatment show more signs and symptoms of TMD than untreated individuals, but is in contrast to various other studies that do not support a causative relationship between orthodontics and TMJ problems [47].

For this reason longitudinal studies are needed to clarify these issues.

The present work has limitations related to sampling and analysis in that electromyographic data were not available for Study group subjects before or during the treatment. Ideally, a study should be conducted that follows patients over several years starting from the original diagnosis, ultimately comparing data between those that received orthodontic treatment and those that did not. This type of longitudinal study is in progress in our clinic, but the results will not be available for several more years. A further limitation is related to the ongoing debate over the value of sEMG at rest. Although some authors have claimed such data do not have a sufficient signal-to-noise gain to be considered reliable, sEMG remains the only instrument available that can monitor variations in the muscles electric tone. Furthermore, previous works [48,49] have demonstrated that sEMG can be useful in the assessment of neuromuscular patterns.

## Conclusion

At present, the effects of orthodontic treatment on stomatognathic system function are not well understood. sEMG allows one to delineate the neuromuscular patterns of patients, which can be used to define an appropriate orthodontic therapy that takes into consideration the balance of the stomatognathic system. Here, the sEMG activity of anterior temporalis muscles was found to be significantly higher in treated patients than untreated patients. Hence, it is our view that further studies, employing a correct research methodology, more detailed design and larger number of samples are needed to examine the effects that orthodontic treatment, by way of changing occlusion, might have on muscular and articular function. To do so, it will be necessary to use valid diagnostic instruments such as sEMG, which, through the study of patients’ electromyographic parameters, allow the effects of dental changes on the neuromuscular system to be monitored.

## Competing interests

The authors declare that they have no competing interests.

## Authors’ contributions

All authors contributed towards the conception and design of the study and critical revision for important intellectual content. CM, IC and AS contributed with the acquisition, analysis and interpretation of data, AS and MCM contributed scientifically to the paper by performing literature searches, CM, IC and AM contributed with drafting of the manuscript. SN provided statistical support. AM contributed with the design, interpretation of data and revision of the manuscript. All authors read and approved the final manuscript.

## Pre-publication history

The pre-publication history for this paper can be accessed here:

http://www.biomedcentral.com/1472-6831/13/57/prepub
